# Cross Talk of Proteostasis and Mitostasis in Cellular Homeodynamics, Ageing, and Disease

**DOI:** 10.1155/2016/4587691

**Published:** 2016-02-09

**Authors:** Sentiljana Gumeni, Ioannis P. Trougakos

**Affiliations:** Department of Cell Biology and Biophysics, Faculty of Biology, National & Kapodistrian University of Athens, 157 84 Athens, Greece

## Abstract

Mitochondria are highly dynamic organelles that provide essential metabolic functions and represent the major bioenergetic hub of eukaryotic cell. Therefore, maintenance of mitochondria activity is necessary for the proper cellular function and survival. To this end, several mechanisms that act at different levels and time points have been developed to ensure mitochondria quality control. An interconnected highly integrated system of mitochondrial and cytosolic chaperones and proteases along with the fission/fusion machinery represents the surveillance scaffold of mitostasis. Moreover, nonreversible mitochondrial damage targets the organelle to a specific autophagic removal, namely, mitophagy. Beyond the organelle dynamics, the constant interaction with the ubiquitin-proteasome-system (UPS) has become an emerging aspect of healthy mitochondria. Dysfunction of mitochondria and UPS increases with age and correlates with many age-related diseases including cancer and neurodegeneration. In this review, we discuss the functional cross talk of proteostasis and mitostasis in cellular homeodynamics and the impairment of mitochondrial quality control during ageing, cancer, and neurodegeneration.

## 1. Introduction

Cells express a pool of thousands of different proteins that need to be tightly controlled for proper cellular structure, organization, and function. The proteostasis network (PN) is an assembly of distinct dynamic molecular pathways that control the functionality of the proteome (proteome homeodynamics) during protein synthesis, folding, trafficking, and degradation. Failure of the PN is associated with broad range of diseases including cancer, neurodegeneration, and immunological and metabolic disorders [1]. Ageing leads to a gradual dysfunction of the proteostasis network and thus to proteome instability due to accumulation of damaged and/or misfolded proteins [2].

Mitochondria are the energy producing organelles in eukaryotic cell providing ATP through oxidative phosphorylation (OXPHOS). Moreover, mitochondria control cell death through apoptosis and supply Ca^2+^ and metabolites required for cellular homeodynamics [3]. We propose the term homeodynamics (instead of the term homeostasis) since cellular functionality obviously reflects a delicate highly dynamic balance of different (usually opposing in their final output) molecular pathways that aim towards a preset ideal equilibrium status rather than a static condition which is the true meaning of the word “-stasis” (from Greek *στάσις* “standing still”). In other words, the term homeostasis fails to illustrate the dynamic, adapting, and thus constantly remodelling nature of biological systems which determines survival (see also Rattan, 2014 and Demirovic and Rattan, 2013) [4, 5].

In line with this notion, mitochondria are highly dynamic organelles that undergo fission and fusion and move into the cell along the microtubules to generate the mitochondrial network [6]. Proper mitochondrial function also determines the functionality of most (if not all) of the other cellular organelles because of the specialized interacting functional networks that are generated; part of these networks is also established by contacts of the mitochondria with organelles (e.g., with endoplasmic reticulum, plasma membrane, and peroxisomes) [7–9]. For instance, the association of mitochondria with the endoplasmic reticulum (ER), in a juxtaposition known as Mitochondria-Associated Membrane (MAM), has an important role in controlling mitochondria biogenesis, Ca^2+^ release, and lipid synthesis and apoptosis [10, 11]. In addition, the subcellular distribution of mitochondria can affect the cellular transcriptome and transcription rates. A recent study showed that mitochondria clustering around the perinuclear region can act as signaling for increased oxidative stress affecting hypoxia inducible promoters [12].

Mitochondrial dysfunction has also been associated with ageing and most of the so-called age-related diseases [13–17]. The maintenance of “healthy” and fully functional mitochondria is thus essential for cellular homeodynamics. A first check point and active surveillance is provided by the organelle itself. The mitochondria have their own chaperones and proteolytic enzymes that remove damaged or unfolded proteins [18–20]. Furthermore, impaired mitochondrial function and instability of the mitochondrial proteome activate a specific ubiquitin-proteasome response known as mitochondrial UPR (UPR^mt^); UPR^mt^ thus provides a link between mitochondrial survival pathways and the multitasking UPS.

The plasticity of the mitochondria allows continuous changes of their shape and number, while their morphology is maintained by the equilibrium of fusion and fission events. Mitochondria undergo fusion and fission in order to avoid damage accumulation or respond to certain bioenergetics demands [21]. Fusion rearranges the matrix content of a damaged mitochondrion with a healthy one, diluting thus mutated DNA copies and unfolded proteome [22]. On the other hand, fission is important for mitochondria division and elimination of damaged mitochondria by autophagy [23]. If an extensive mitochondria damage persist the cells fate the apoptosis pathway releasing proapoptotic factors [24].

Herein, we will focus on cross talk of proteostasis and mitostasis in cellular homeodynamics, ageing, and disease.

## 2. Mitochondrial Chaperones and Proteases: Repair/Refold and Recycle

### 2.1. Chaperones

**Figure 1:**
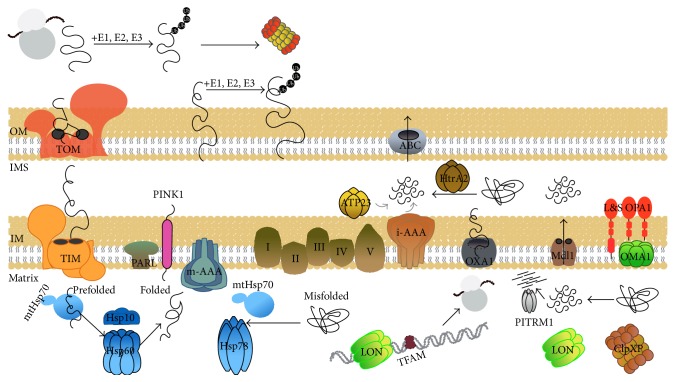
Mitochondrial quality control by molecular chaperones and proteases. Mitochondrial precursors synthesized in cytosol are imported in the mitochondrial matrix via the TOM and TIM translocases. Misfolded precursors are degraded by the 26S proteasome in the cytosol before they enter mitochondria; the 26S proteasome also degrades (following ubiquitination) proteins of the outer mitochondria membrane (OM). Precursors imported in the mitochondrial matrix are bound to chaperones (e.g., mtHsp70 and Hsp60/Hsp10) which then drive their proper folding; mtHsp70 along with Hsp78 also promote protein disaggregation during stress conditions. The polypeptides of the respiratory complex protein machines which are encoded by either mtDNA or genomic DNA are transported into the inner membrane (IM) by the Oxa1 peptide transporter. Damaged and/or unfolded matrix proteins are degraded by the LON, ClpXP, and m-AAA proteases, while the generated peptides can be further degraded by PITRM1; LON protease also degrades the TFAM transcription factor. Peptides generated by the ClpXP protein are transported across the inner mitochondrial membrane by the matrix ATP-dependent peptide transporter HAF-1 (Mdl1 in yeast). The PINK1 protein is encoded at the genomic DNA and after being transported at the IM it is processed by PARL. In the case of mitochondrial dysfunction or damage PINK1 translocates at the OM and facilitates the activation of autophagy/mitophagy machinery (see text). Similarly to PINK1, OPA1 is imported from the cytosol and is processed in long (L) and short (S) isoforms which are located at the IM and the intermembrane space (IMS), respectively. During mitochondrial dysfunction OPA1 isoforms are processed by OMA1 (and, likely, PARL), while unfolded, misfolded, and/or damaged proteins of the IMS are processed by the HtrA2 and i-AAA proteases; generated peptides are then released in the cytosol by the ATP binding cassette transporter (ABC transporter). Mitochondrial inner membrane protease ATP23 is thought to participate in the maintenance of the respiratory chain; however, its role still remains to be elucidated. Mentioned molecules along with their relative topologies and processing (arrows) are indicated in the figure.

The mitochondrial proteome is composed of ~1500 peptides, of which only 13 are encoded by the mitochondrial genome. Therefore, the vast majority of mitochondrial proteins are synthesized in the cytosol and must be imported into the organelle [25, 26]. Most of the matrix proteins are transported in the mitochondria as precursor proteins, which are subsequently cleaved and assembled in multiprotein complexes (which can also be viewed as complex protein machines). Precursor proteins are transported across the narrow pores formed by the Translocase of the Outer Membrane (TOM) and the Translocase of the Inner Membrane (TIM) complexes, mostly in an unfolded state [27]. The whole process is under the surveillance of molecular chaperones in order to avoid the formation of protein aggregates or misfolded proteins (Figure 1). The nascent precursor peptide is bound by the cytosolic Hsp70 and Hsp90 chaperones that protect the hydrophobic segments of the peptide and keep them in unfolded conformation [28]. After the translocation in the mitochondria, the precursor peptide is bound to the matrix chaperones.

The two most dynamic networks of mitochondria chaperones are the mtHsp70 (an Hsp70 family member) and the multimeric Hsp60-Hsp10 machineries [29]. The mtHsp70 is part of the presequence translocase-associated import-motor (PAM) complex, which directly folds the incoming proteins. The mtHsp70 (via an ATP-dependent process) guides the translocation of the polypeptide chain through the translocase complexes of the outer and inner mitochondrial membranes and its complete unfolding [30].

Hsp60 forms large tetradecameric protein complexes consisting of two stacked rings that allow the accommodation of the unfolded polypeptide. The cavity of each ring gets closed by the Hsp10 cofactor. Conformational changes, after hydrolysis of ATP, lead to a more hydrophilic cavity which allows the folding of the polypeptide. The newly folded protein is then released after opening of the ring cavity by the dissociation of Hsp10 [31]. Hsp60 is required for the folding of new precursor peptides and plays an essential role in mitochondrial protein biogenesis [32].

An additional chaperone is Hsp78 (a member of the ClpB/Hsp104 family) which has a disaggregation function under stress conditions; Hsp78 is essential for the respiratory chain reaction and mitochondrial genome integrity under severe stress [33]. Mitochondrial chaperones deletion in yeast has lethal effects, indicating that heat shock proteins have an essential role in mitochondria quality control and protection of the organelle from unfolded protein aggregates and proteome instability [34].

#### 2.1.1. The Hsp90-Type Chaperone TRAP1

TRAP1, also known as Hsp75, was initially identified as an Hsp90-like chaperone that interacts with the tumor necrosis factor (TNF) receptor and the retinoblastoma protein (Rb) [35]. However, later studies revealed that TRAP1 localizes in the mitochondrial matrix of mammalian cells [36, 37]. TRAP1 exhibits a significant sequence and structure similarity to the members of the Hsp90 family; these chaperones have a mitochondrial targeting sequence at their N-terminus (which is cleaved after mitochondrial translocation) and an ATP binding domain. The ATP binding site is the most conserved region between Hsp90 and TRAP1 [35, 38]. TRAP1 shows different functional characteristics from other chaperones and its expression in the cytosol could not rescue the Hsp90 loss of function phenotypes [35]. TRAP1 is thought to also play an important role in preventing cell death due to ROS accumulation. Specifically, downregulation of TRAP1 leads to ROS accumulation, while its overexpression suppresses ROS production [39, 40]. Moreover, TRAP1 regulates metabolic switch between oxidative phosphorylation and aerobic glycolysis [41]. Loss of TRAP1 in immortalized mouse fibroblasts and in human tumor cells resulted in increased mitochondrial respiration, as well as in increased oxygen consumption and ATP levels; these phenotypes were associated with suppression of aerobic glycolysis [41]. Further studies have shown that TRAP1 interacts with cyclophilin D and regulates the mitochondrial permeability transition pore to suppress cell death [42]. In addition, TRAP1 seems to promote neoplastic growth by inhibiting succinate dehydrogenase and downregulating cell respiration in colon carcinoma cells. It was reported that OXPHOS deregulation stabilizes the transcription factor HIF1*α* promoting tumor growth [43]. Also, it was found that TRAP1 is phosphorylated by PINK1 protein (see below) to promote cell survival [44]. Because of its cell protective role and since both the mRNA and proteins levels of TRAP1 are highly expressed in certain cancer cell lines and tumors, TRAP1 has been proposed as an anticancer therapeutic target [45, 46]. To this end, Gamitrinibs are the first mitochondria-targeted molecules which inhibit Hsp90 and TRAP1 and induce mitochondrial membrane permeabilization [45, 47]. Nevertheless, expression of TRAP1 in cancer cells is variable and in some cancers TRAP1 is even downregulated as compared to normal tissue counterparts [48, 49]. Therefore, further studies are needed to unequivocally demonstrate the role of TRAP1 in tumorigenesis.

### 2.2. Proteases

The mitochondrial respiratory chain is one of the main sources of endogenous reactive oxygen species (ROS). Generated ROS can oxidize (among others) the mitochondrial proteins and lead to accumulation of damaged and/or misfolded proteins [50, 51]. Therefore, loss of function proteins due to exposure to oxidative stress must be either* fold, hold, or degrade*; these options are mostly guided by the action of chaperones, since unfolded proteins that overcome the capacity of chaperones for refolding need to be removed by alternative pathways. The turnover of unfolded or damaged proteins is driven by a complex network of mitochondrial proteases that collaborate for this task with mitochondrial chaperones [52]. There are (a) the ATP-dependent proteases, namely, the LON protease and the Clp Protease Proteolytic subunit (CLPP) and the mitochondrial AAA (ATPases Associated with diverse cellular Activities) proteases of the inner mitochondrial membrane and matrix; (b) the two ATP independent proteases, the ATP23 and HtrA2; and (c) two oligopeptidases, namely, the presequence protease (PITRM1, also known as PreP) and the mitochondrial oligopeptidase M (MEP, also known as neurolysin) [53] (Figure 1).

#### 2.2.1. LON Protease

The LON protease, firstly identified in bacteria as La protein [54], is conserved among prokaryotes and eukaryotes. LON protein is encoded by the nuclear genome and belongs to the AAA+ protein family. This protease contains three domains of different functions: the N-terminal domain that interacts with protein substrates together with the second AAA+ domain (being involved in ATP binding and hydrolysis) and a third domain bearing the catalytic and proteolytic activity, respectively [55]. LON has a typical serine-lysine dyad at the active center and acts as homooligomeric complex of seven monomers in eukaryotes [56]. LON degrades oxidized and damaged proteins in association with chaperones which maintain the protein in unfolded state until the initiation of the proteolytic reaction [57]. Although the recognition mechanism of the target protein by LON still remains to be elucidated, it is thought that important features must be the overall structure of the protein and the exposed loops at the surface of substrate [58].

Notably, LON activity is not limited to misfolded and/or damaged proteins since several other proteins have been identified as LON substrates under normal conditions, including succinate dehydrogenase subunit 5, glutaminase C, cystathionine *β*-synthase, and cytochrome *c* oxidase subunit 4 isoform 1 [59–62].

Finally, LON protease has been associated with mitochondrial DNA regulation. LON binds to mitochondrial DNA and regulates mitochondrial DNA copy number and transcription by targeting the mitochondrial transcription factor A (TFAM) for degradation [63, 64]. Loss of the LON yeast homolog, PIM1, resulted in a respiratory deficient phenotype, whereas loss of LON function in human lung fibroblasts enhanced apoptosis and altered mitochondria morphology [65, 66]. Moreover, deficiency of LON in a mouse model showed alteration of OXPHOS and of mitochondrial respiratory chain activity [67].

Several experiments have shown the functional involvement of the LON protein in ageing, as well as in tumorigenic transformation [68, 69]. More specifically, LON overexpression increased lifespan and healthspan in* Podospora anserina*, while aged mice presented decreased protein levels of LON; these effects associate with oxidatively damaged mitochondrial proteins and mitochondrial dysfunction [66, 69]. Nevertheless, further studies are required to better clarify the functional involvement of LON in cancer and ageing, as well as in programmed cell death.

#### 2.2.2. ClpP Protease

The ClpP protease is a large oligomeric protein complex being conserved from bacteria to higher eukaryotes [70, 71]. The proteolytic core of ClpP protease is formed of two stacked rings with 7 subunits each. ClpP is activated after the formation of a complex with ClpX, an AAA chaperone protein in the mitochondrial matrix; the chaperone partner component is involved in the initial recognition of the substrate polypeptide, its unfolding in an ATP-dependent way, and its translocation into the proteolytic chamber of the ClpP complex [72]. ClpP protease lacks homolog in yeast, but intriguingly it has identified a homolog for the ClpX chaperone, namely, the Mcx1 protein. However, deletion of Mcx1 in yeast did not show any prominent phenotype [73], while ClpP null mice demonstrate loss of fertility, failure of hearing and accumulation of ClpX and mtDNA [74]. Studies in human mitochondria have shown that there is a correlation of increased ClpP protein levels with the amount of mutated and unfolded proteins in mitochondria, suggesting a decisive role of this protease in the mitochondrial UPR response [75].

#### 2.2.3. The Fts-H Type, AAA Proteases

The LON and ClpP proteases are soluble enzymes and therefore have no access to the membrane proteins or the proteins located in the intermembrane space. Thus, for the membrane protein quality control, mitochondria have separate proteolytic enzymes dedicated to proteolysis of membrane-integrated substrate proteins. These enzymes are mitochondrial AAA (ATPases Associated with diverse cellular Activities) proteins that belong to the filament-forming temperature-sensitive (Fts-H) protease family, named after the bacterial founding member. Members of this family have a zinc metalloprotease domain, a regulatory domain belonging to the AAA family, and a transmembrane domain [76, 77]. Two type members with different membrane topologies have been described, namely, the i-AAA members which expose their catalytic site in the intermembrane space and the m-AAA which expose their catalytic sites in the mitochondrial matrix.

The i-AAA proteases are involved in the degradation of nonassembled proteins in the intermembrane space [78]. Misfolded and/or mutated proteins are degraded to peptides and are further exported from the organelle or degraded to amino acids by various oligopeptidases.

A mitochondrial m-AAA protease was first described in yeast as a heterooligomeric complex composed of highly homologous subunits (Yta10p and Yta12p). The human m-AAA counterpart protease is composed of paraplegin and AFG3L2 [79, 80] which in human cells exist in two isoforms; one which forms an oligomer with paraplegin and another which forms homooligomers [81].

The AAA proteases have an important role in the proper assembly of the respiratory chain enzyme complexes [82]. Specifically, the biogenesis and assembly of the mitochondrial respiratory complexes is a complicated operation of proteins encoded by both nuclear and mitochondrial DNA, and thus the chances for accumulation of nonassembled subunits in the membrane increase. It is worth mentioning that the substrates of m-AAA protease in yeast are not only the respiratory complex components but this protease has a functional role in mediating proteolytic maturation of additional proteins [78], such as the mitochondrial ribosomal component MrpL32; this ribosomal component is also processed by the m-AAA human isoforms [83]. The m-AAA protease paraplegin AFG3L2 is also involved in OPA1 processing together with Presenilin-Associated Rhomboid-Like (PARL) protease in generating OPA1 isoforms [84]. Interestingly, these are not the only proteases being involved in OPA1 cleavage which is also cleaved by Yme1L, an i-AAA protease anchored in the inner membrane which affects generation of OPA1 isoforms [85].

#### 2.2.4. The HtrA2 Protease

The HtrA2 protease is conserved in animals and plants but not in yeast. HtrA2 consists of a serine protease domain and a PDZ domain involved in substrate binding and regulation of the enzymatic structure. Notably, this protease has the interesting ability to switch between protease and chaperone activity based on the temperature. Under normal conditions, HtrA2 acts mostly as a chaperone, but in stress conditions (e.g., due to temperature increase) HtrA2 exerts proteolytic activity and degrades the nonfunctional proteins [86]. Similarly, in bacteria, the HtrA2 homolog (HtrA/DegP) has a protein quality control role in the periplasmic space at elevated temperatures [87].

The human HtrA2/OMI localizes in the mitochondrial intermembrane space and its expression levels are increased during stress conditions [88]. Loss of HtrA2 increases the number of damaged mitochondria and of the unfolded respiratory chain subunits. Moreover, it was found that HtrA2 associates with programmed cell death, as well as with necrosis [89]; specifically, HtrA2 is released in the cytosol during apoptosis and cleaves antiapoptotic proteins [90, 91]. On the other hand, it was reported that HtrA2 is linked to the mitochondrial inner membrane and is being activated by PARL cleavage to prevent accumulation of proapoptotic proteins in the outer membrane [92]. HtrA2 has also been associated with alterations of mtDNA as loss of HtrA2 in mouse cells leads to accumulation of mtDNA mutations [93].

HtrA2 functionally interacts with the mitochondrial protein kinase PINK1 and mouse models lacking HtrA2 develop neurological defects reminiscent of Parkinson's Disease [94, 95]. HtrA2 knockout mice have decreased mitochondrial membrane potential and display mitochondrial uncoupling [96]. In addition, loss of HTRA2 results in ATP depletion and reduced mitochondrial mass [96]. Finally, studies in mice have revealed an implication of HtrA2/OMI in ageing [97]. Therefore, loss of HtrA2/OMI relates to both premature ageing and neurodegeneration.

#### 2.2.5. The PITRM1 Proteases

PITRM1 is a highly conserved zinc metalloprotease known also as Presequence Peptidase (PreP). PITRM1 was identified in* Arabidopsis thaliana* as a protease that degrades targeting peptides in both mitochondria and chloroplasts [98]. PITRM1 localizes in the mitochondrial matrix and is involved in the cleavage of mitochondrial targeting peptides as well as unstructured peptides [98]. Human PITRM1 is a metalloendoprotease of the pitrilysin family [99], which is thought to have a role in mitochondria quality control with a broad range of predicted substrates. In humans, PITRM1 has been implicated in Alzheimer's Disease having a principal role in the degradation of the amyloid *β*-peptides [99] which inhibit peptide turnover and promote the accumulation of nonprocessed preproteins within mitochondria [100]. Incomplete processing of mitochondrial preproteins leads to their destabilization and accelerated turnover [101].

## 3. A Close Network with UPS

### 3.1. The UPS System

The proteasome is a large complicated protein machine of about 2.5 MDa. The 26S proteasome consists of the 20S core particle (CP) and the 19S regulatory particle (RP) [102, 103]. The 20S CP in eukaryotes consists of 28 *α*-type and *β*-type subunits organized in four rings [104]; it carries the catalytic center with the three peptidase activities, namely, the caspase-like, trypsin-like, and chymotrypsin-like peptidase activities [105, 106]. The 19S RP consists of 20 conserved subunits that form the two subcomplexes, known as the base and the lid [102, 107–109]. The lid is composed of nine non-ATPase subunits (Rpn3, Rpns5–9, Rpn11, Rpn12, and Rpn15), while the base is composed of six AAA-type ATPases (Rpt1–6) and three non-ATPases, namely, the Rpn1, Rpn2, and Rpn13 subunits [108, 110–113]. Proteasomes are mainly found in the nucleus and the cytosol [114].

UPS is responsible for the ATP-dependent degradation of either normal short-lived ubiquitinated proteins or misfolded, unfolded, and/or damaged proteins [115]. Ubiquitin (Ub), is a small 76 amino acid polypeptide that is attached to proteins as either a monomer or as a polyubiquitin chain by an enzymatic reaction; Ub is conserved among the eukaryotes but not in prokaryotes [115]. Notably, a small protein, known as prokaryotic ubiquitin-like protein (Pup), has been described in* Mycobacterium tuberculosis*; Pup modifies proteins posttranslationally for proteasome degradation. Pup contains an ubiquitin-like Gly-Gly motif, binds covalently the lysines residues, and targets proteins for proteolysis [116].

The conjugation of Ub to the polypeptide is orchestrated by a series of enzymes (ligases) known as Ub-activating enzymes (E1, E2, and E3). The E1 and E2 enzymes activate the ubiquitin in an ATP-dependent process, while the E3 ligase performs the final step ligating the carboxyl group of the C-terminal of Ub to the target protein [117]. Degradation of the targeted protein by (mainly) the proteasome requires polyubiquitination at lysine 48. However, ubiquitylation is also used for other cellular processes such as immune responses, protein endocytosis, DNA repair, or the assembly of signaling complexes [118, 119]. Proteasome localizes principally in nucleus and cytosol, while proteasome genes are also regulated in a tissue-specific manner during ageing and dietary restriction in liver and brain [113]. In support, studies of our group, and others, have shown the differential* in vivo* regulation of proteasome genes expression and proteasome peptidase activities in somatic tissues and gonads [120, 121].

### 3.2. UPS and Mitochondria

Mitochondrial outer membrane proteins have an important role in the regulation of metabolism, mitochondrial morphology, apoptosis, protein import into mitochondria, and other signaling pathways. Therefore, the maintenance of the outer membrane protein quality control is essential for the organelle function. A number of ubiquitin ligases have been localized on the mitochondrial outer membrane including MULAN, MARCH-V/MITOL, and Mdm30. These ligases affect mitochondrial dynamics by ubiquitinating the proteins being involved in mitochondria fusion and fission processes [122–125]. Notably, no specific mitochondrial proteases have been identified at this compartment.

Several lines of evidence indicate the involvement of cytosolic UPS in mitochondrial outer membrane protein regulation and recycling during proteotoxic stress [126–128]. Mitochondrial Unfolded Protein Response (UPR^mt^) induces outer mitochondrial membrane-associated degradation (OMMAD) and/or mitophagy or even apoptosis if the disruption of mitostasis and/or mitochondrial proteome stability is irreversible [20].

In addition, a role of proteasome in the biogenesis of precursor proteins and in controlling mitochondrial proteome fate has been proposed. Treatment of cells with MG132, a specific proteasome inhibitor, stabilized the precursor forms of OPA1 [129], while intramembrane space proteins that utilize the mitochondrial oxidative folding pathway (MIA pathway) can be ubiquitinated and degraded by the proteasome before they arrive at the mitochondria [130].

In yeast, Fzo1 (Mitofusin ortholog) degradation is mediated by the 26S proteasome [125]. Likewise, Mitofusin 1 (Mfn1) and Mitofusin 2 (Mfn2) (both involved in mitochondrial fusion; see below) are substrates of the UPS [131]. More specifically, after Parkin-mediated ubiquitination, both Mfn1 and Mfn2 can be degraded in a proteasome- and Vms1-p97/CDC48-dependent manner [132, 133]; Vms1-p97/CDC48 is an ubiquitin-selective chaperone that unfolds proteins and disassembles protein complexes and it is thought to play an important role in mitochondria quality control [134]. Vms1 localizes primarily to the cytosol but under stress conditions translocates to the mitochondria through its mitochondrial targeting domain and provides the main driving force for outer mitochondria protein extraction [135]. Furthermore, the association of four deubiquitinating enzymes (DUBs) (that drive an opposite to E3 ligases function) with mitochondria has been described. The Usp9x, Usp30, Usp36, and ataxin-3 may preserve mitochondrial protein degradation by editing or removing the degradative ubiquitin signal [113, 136–138]. However, further studies are needed to clarify which are the sensors of the OMMAD response and the detailed role of the UPS in mitochondria quality control. Given the multiplicity of enzymes involved and their differential subcellular localization it is essential to understand how these enzymes work together and regulate these processes.

Interestingly, additional evidence suggests a role of UPS not only in controlling the outer membrane protein quality but also in the regulation of the proteome of other mitochondrial compartments, such as the matrix [oligomycin sensitivity-conferring protein (OSCP), component of the mitochondrial membrane ATP synthase], the intramembrane space (Endonuclease G), and the inner membrane [Uncoupling Protein-2 and Uncoupling Protein-3 (UCP2 and UCP3)] [139–141]. Nevertheless, the exact mechanism of how UPS mediates the degradation of the inner mitochondria compartments proteins is still elusive and thus further studies are needed to define if and how these proteins are transported at the mitochondria outer membrane or if the UPS can directly access these compartments.

### 3.3. UPR^mt^: A Mitochondria Specific Unfold Protein Response

The UPR^mt^ was firstly described in mammalian cells as a mitochondrial stress response. Depletion of mtDNA or overexpression of a nuclear-encoded aggregation-prone protein in mitochondrial matrix induced increased gene expression of the mitochondrial molecular chaperone Hsp60 and of the protease ClpP [142, 143]. Although UPR^mt^ has been studied in different model organisms,* C. elegans* has been a useful model for the comprehension of this pathway. The first described component of the UPR^mt^ is the C/EBP homology protein (CHOP). CHOP heterodimerizes with C/EBP*β* and by binding to the promoter region of Hsp60 increases its transcription levels [60]. Further analysis of CHOP and C/EBP*β* revealed that these proteins contain at their promoter region two additional conserved sequences, known as conserved Mitochondrial Unfolded Response Elements (MUREs) [144]. Activation of CHOP is not specific for mitochondrial stress but can also relate to ER stress conditions or even exposure to arsenate [145, 146].

Using a genome-wide RNAi screening various mediators of the UPR^mt^ have been identified. Accumulated unfolded proteins are processed by the ClpXP protein and transported across the inner mitochondrial membrane by the matrix ATP-dependent peptide transporter HAF-1 (Mdl1 in yeast) [147–149]. Deletion of ClpXP disrupts the proteolysis of unfolded mitochondrial proteins, whereas deletion of HAF-1 attenuates its activation during stress [148]. Both proteins are essential for the survival and normal lifespan during mitochondrial stress condition, underlying the important role of ClpXP and HAF-1 in mitochondria quality control. Another downstream component of HAF-1 is the bZip transcription factor ATFS-1 (Activating Transcription Factor associated with Stress). Under normal conditions, ATFS-1 is imported in mitochondria and degraded by the LON protease [150]. During mitochondrial stress ATFS-1 accumulates in the nucleus and activates transcription of UPR^mt^ genes [151]. Deletion of ClpP and HAF-1 prevented nuclear accumulation of ATFS-1 underlying its downstream activation in a HAF-1 dependent manner [147, 151]. DVE-1/UBL-5 is a protein complex that is necessary for the activation of UPR^mt^ response and acts downstream of ClpXP/HAF-1. DVE-1 is a conserved transcription factor that binds to Hsp60 promoter, while UBL-5 is an ubiquitin-like protein that is upregulated and binds to DVE-1 in response to mitochondrial stress [148, 152].

A growing number of studies underlie the involvement of UPR^mt^ in longevity. Specifically, reduction of the* C. elegans* NAD^+^ levels decreased lifespan, while rescue experiments involving the protein deacetylase sir-2.1 (NAD-dependent enzyme) and activation of UPR^mt^ prevented the associated metabolic decline and extended lifespan [153]; in these experiments overexpression of deacetylase sir-2.1 induced lifespan extension in an UPR^mt^-dependent manner. Furthermore, silencing of CCO-1, a subunit of Cytochrome *c* oxidase, in* C. elegans*, increased lifespan and induced UPR^mt^ [154]. Ribosomal protein S5 (Mrps5) was described as a candidate gene that regulates mouse lifespan. Knockdown of Mrps5 in worm increased lifespan and prompted activation of UPR^mt^ [155]; notably, however, UPR^mt^ activation does not always induce lifespan extension [156].

Finally, as the components of the UPR^mt^ response are important for cell survival, many tumors and cancer cell lines display an accumulation of unfolded proteins and activated UPR^mt^ response [157, 158]. Nevertheless, the exact mechanism of the UPR^mt^ response in longevity and disease and what factors determine its activation in each case still remain to be elucidated.

## 4. Mitochondria Dynamics: Mix and Segregation

**Figure 2:**
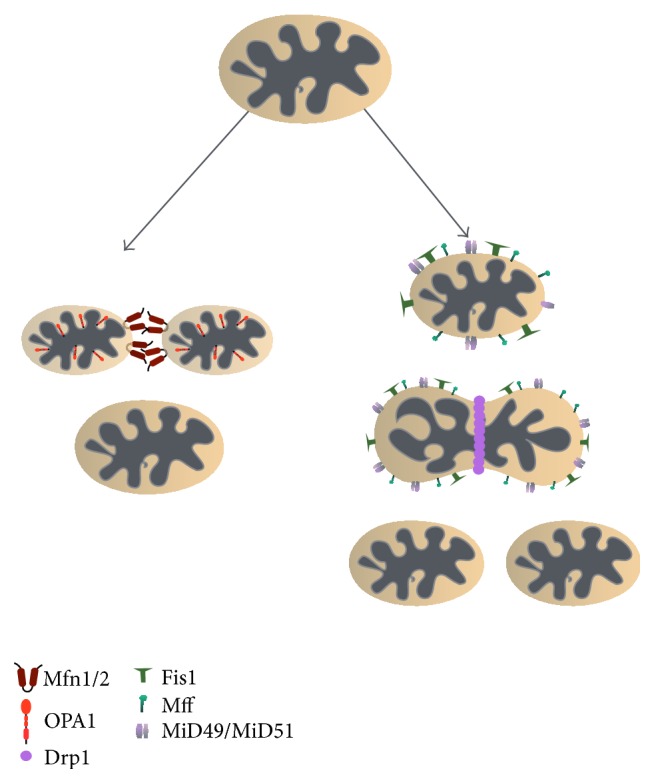
Mitochondrial dynamics. Mitochondrial morphology and cellular network are regulated by continuous balance and dynamic regulation of fusion and fission events. Fusion is mediated by the Mitofusin 1 (Mfn1) and Mitofusin 2 (Mfn2) GTPases of the OM, as well as from OPA1 of the IM (see Figure 1 for abbreviations). Mfn1 and Mfn2 promote fusion (via the interaction of their coiled-coil domains) of the OMs of two juxtaposed mitochondria and this event is followed by OPA1-mediated fusion of the IMs (left arrow). On the other hand, fission generates two daughter organelles from a mitochondrion. Drp1 is recruited to the mitochondria OM where it directly interacts with Fis1, Mff, and MiD49/MiD51. Then Drp1 generates a ring structure that constricts the mitochondrial membranes leading to the formation of two daughter mitochondria (right arrow).

When the molecular pathways of chaperones and proteases are overwhelmed additional quality control mechanisms concerning the entire organelle homeodynamics are activated. Specifically, mitochondria undergo continuous cycles of fusion and fission in order to dilute damage. Both processes are regulated by a number of GTPases (guanosine triphosphatases) conserved from yeast to mammal (Figure 2). The importance of the fusion and fission events is highlighted by a number of disorders caused by mutations of the proteins involved in such processes (see below). Since mitochondria are double membrane organelles, fusion and fission processes involve proteins localized on both compartments.

### 4.1. Fission

Fission is an important process for the generation of new daughter mitochondria; this event is mainly driven by the dynamin-related protein 1 (Drp1). Drp1 is a cytosolic protein that translocates at the mitochondrial outer membrane to initiate the fission process. Once localized in the outer membrane, Drp1 is oligomerized into a spiral-like structure and constricts the outer and inner mitochondria membrane by inducing high curvature in a GTP hydrolysis-dependent way [159]. Fission is tightly regulated by several posttranslational modifications of Drp1. The first described is the phosphorylation by Cdk1/cyclin B which enhances mitochondrial fragmentation during mitosis [160]. Fission may also be inhibited by kinase A-mediated phosphorylation of Drp1 at Serine 637, a highly conserved Drp1 amino acid at metazoans. Phosphorylation at Ser^637^ inhibits GTPase activation of Drp1 and, likely, the recruitment of Drp1 to the outer membrane [161]. Other posttranslational events of Drp1, like nitrosylation and sumoylation, promote mitochondrial fission [162, 163]. Drp1 is also target of the ubiquitin ligase MARCH5/MITOL; in this case ubiquitination of Drp1 by this ligase does not target Drp1 for degradation but rather regulates the formation of membrane complexes and protein activity [164].

Recruitment of Drp1 to the mitochondrial membrane is mediated by receptor proteins. Specifically, the yeast homolog of Drp1 (Dnm1p) is recruited by the receptor protein Fis1p [165]. In line with this finding, overexpression of Fis1 in mammalian cells promotes fission; however, its downregulation does not affect this process [166]. In eukaryotes, other interaction factors, like Mff, MiD49, and MiD51/Mief1, have been proposed to be functionally involved in Drp1-mediated fission [167–169]. The large number of factors which contribute to tight regulation of Drp1 function clearly highlights the importance of the fission event for mitochondria homeodynamics.

### 4.2. Fusion

During fusion, mitochondria mix their genetic content in order to complement deficit of damaged mitochondria. In contrast to fission, mitochondrial fusion is operated by three dynamically related GTPases proteins, namely, Mfn1, Mfn2, and Optic Atrophy 1 (OPA1). Mfn1 and Mfn2 are implicated in the fusion of the mitochondrial outer membrane, whereas OPA1 is involved in the fusion of the inner membrane [170, 171]. Mfns were firstly described in* Drosophila melanogaster* [fuzzy onions, (Fzo)]; Mfn homologs were later on also described in yeast (Fzo1) and in mammals (Mfn1 and Mfn2) [172, 173]. Mechanistically, the Mfn1 and Mfn2 proteins tether the outer membrane of the mitochondria by forming homo- and heterooligomers [174]. Downregulation of Mfn1 or Mfn2 in cells leads to mitochondrial fragmentation; additionally, lack of either Mfn1 or Mfn2 implies the total loss of fusion, evidencing that both proteins are essential for this mitochondrial process [170].

OPA1 is a conserved large GTPase of the dynamin family, imported at the mitochondrial membrane by its N-terminal sequence. Opal is involved in cristea remodelling and inner membrane fusion [175], while mutations of OPA1 lead to neuropathy of optic nerve known as dominant optic atrophy [176]. This GTPase has different splicing isoforms. Specifically, there are two types of forms, the long (L) form that is membrane anchored and the short (S) form that is found soluble in the intramembrane space [177]. The balance between these two pools of isoforms can regulate the fusion process since reduction of the membrane anchored forms by activation of the metalloprotease OMA1 during either stress conditions or decrease of the mitochondrial membrane potential suppresses the fusion events [178]. On the other hand, oxidative phosphorylation can enhance the mitochondrial inner membrane fusion [179]. Interestingly, loss of OPA1 results in loss of inner membrane fusion but does not affect the fusion of the outer membrane, suggesting that fusion-involved proteins can act in different phases and by distinct modes during this process [70].

### 4.3. Mitochondria Motility

Another important aspect of mitochondria dynamics is their motility and cellular distribution. The role and significance of this process are especially highlighted in neurons which need mitochondria energy at sites distant from the cell body [180]. The transport of the mitochondria is a cytoskeleton based movement [181]. In mammalian axons of neuronal cells, mitochondrial movement from the cell body to the synaptic junctions (known as anterograde movement) is driven by the kinesin-1 motor (KHC, Kif5b) and movement from the synaptic junctions to the cell body (the retrograde movement) is driven by dynein, whereas in yeast the transport is based on actin [182, 183]. The binding of the mitochondria to the kinesin-1 motor is mediated by the adapter proteins Milton and Mitochondrial Rho GTPase (Miro). Milton interacts with kinesin and directly binds to Miro located on the mitochondria outer membrane [184, 185]. Loss of Miro in* Drosophila *resulted in reduction of mitochondria from dendrites and axons [185].

Studies on a knockout mouse model have demonstrated that attachment of the mitochondria to the microtubule can also be regulated by the protein syntaphilin (SNPH). Neuronal depletion of SNPH increased axonal mitochondrial motility, whereas overexpression of SNPH augmented the number of immobile mitochondria [186].

The fusion and fission processes are closely related to the mitochondria motility. Mitochondria fragmentation induced by loss of Mfn1 reduces the mitochondrial motility, while loss of Drp1 in* Drosophila* leads to a decrease of synaptic mitochondria [170, 187]. On the other hand, deletion of Miro in yeast dramatically induces changes in the mitochondrial morphology but seemingly does not affect the fusion or fission processes [188].

## 5. Mitophagy: Remove the Damaged

When a mitochondrial damage or unrepairable dysfunction occurs, selective removal of mitochondria by autophagy takes place; this process is known as mitophagy, a term proposed by Lemasters in 2005 [189].

**Figure 3:**
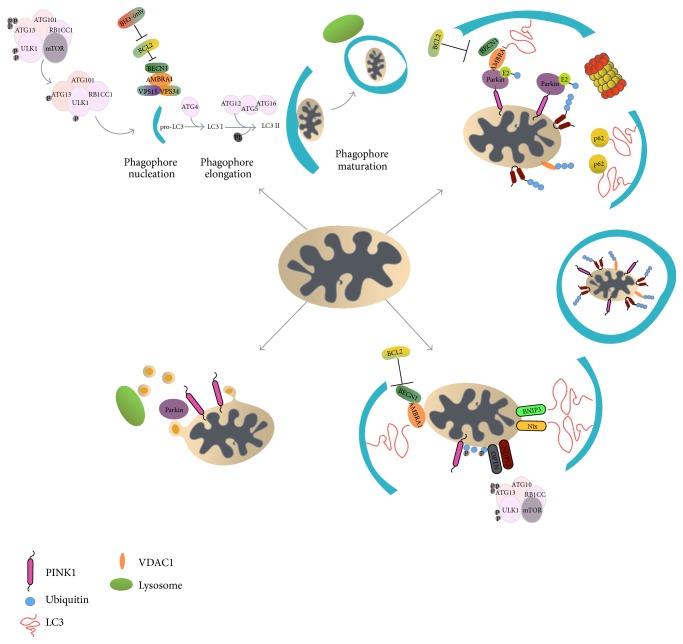
Pathways for the removal of damaged mitochondria. Unrepaired mitochondrial damage or reduced membrane potential (ΔΨ_*m*_) prompts the removal of mitochondria by autophagy. Autophagy starts (upper left) with the upstream complex ULK1 which is composed from Unc-51-Like Kinase 1 protein (ULK1), ATG13, mTOR kinase, and RB1-inducibile Coiled-Coil 1 (RB1CC1). Inhibition of the mTOR kinase leads to the generation of the Beclin1-Vacuolar Protein Sorting (VPS) 34-VPS15 complex. B-cell lymphoma 2 (BCL-2) blocks the induction of autophagy by binding to Beclin1 and to the Activating Molecule in Beclin1-Regulated Autophagy (AMBRA1). Displacement by BH3-only proteins activates Beclin1-VPS 34-VPS15 and induces the phagophore generation. The phagophore is elongated by the autophagy proteins ATG12-ATG5 creating the ATG16L complex, which then conjugates phosphatidylethanolamine (PE) to the procures of microtubule-associated protein 1 Light Chain 3 (LC3) to generate the LC3 II receptor. Finally, the membrane engulfs the cargo, closes its ends, and fuses with lysosomes in order to degrade its content. Mitophagy can also occur in a PINK1/Parkin dependent pathway (lower left; upper right): PINK1 is exposed at the outer membrane, where it recruits the E3 ubiquitin ligase Parkin to mitochondria. Parkin ubiquitinates outer membrane proteins, such as Mfns and Voltage-Dependent Anion Channel (VDAC), which are then degraded by the 26S proteasome. Similarly, p62/SQSTM1 (Sequestosome 1) interacts with ubiquitinated mitochondrial proteins and recruits the autophagosome through its interaction with the LC3 receptor. An alternative PINK1/Park dependent pathway is the formation of cargo-selective vesicles (lower left) which are released from mitochondria (Mitochondria-Derived Vesicles, MDV) and fuse with lysosomes. The formation of MDV is induced by increased ROS levels and does not require mitochondrial depolarization and/or LC3 or ATG5 proteins. Mitophagy in a Parkin-independent way (lower right) may also occur since (a) the autophagy receptors NIX and BNIP3 can directly interact with the autophagosome through the LC3 receptor; (b) AMBRA1 if overexpressed in the mitochondria outer membrane interacts with the LC3 receptor and can induce autophagy by both Parkin dependent and Parkin-independent pathways; and (c) PINK1 phosphorylates the ubiquitin chains in mitochondria promoting the recruitment of NDP52 [also known as Calcium binding and Coil-Coil domain protein 2, (CALCOCO2)] and optineurin autophagy receptors; subsequently, ND52 and optineurin recruit the upstream machinery of autophagy and trigger mitophagy.

Autophagy is an evolutionarily conserved process that is responsible for the lysosome-mediated degradation of cytoplasmic components during a process where an isolated membrane named phagophore is generated upon autophagy signals [190]. The first upstream formed complex of this process in mammalian cells is the ULK1 complex which is composed of the ULK1 (Unc-51-Like Kinase 1 protein), ATG13, mTOR kinase, and the RB1CC1 (RB1-inducibile Coiled-Coil 1). Autophagy induction inhibits mTOR which under physiological conditions is phosphorylated and inhibits the ULK1 and ATG13 proteins of the complex [191, 192]. Phagophore nucleation requires the formation of a complex consisting of the vacuolar protein sorting (VPS) 34, VPS15, Beclin1, and the activating autophagy/beclin-1 regulator 1 (AMBRA1) [193]; in this process, B-cell lymphoma 2 (BCL-2) inhibits autophagy by binding Beclin1, while BCL-2-homology 3 (BH3-only) activates the VPS34 complex by displacement of the BCL-2 protein [194]. Furthermore, the phagophore expands after conjugation of ATG12 to ATG5 which interacts with ATG16 forming the ATG16L complex which then conjugates phosphatidylethanolamine (PE) to the procures of microtubule-associated protein 1 light chain 3 (LC3) until generation of the LC3 II receptor. Expansion of the phagophore continues until its edges surround the cargo, fuse, and form the autophagosome. Finally, the autophagosome fuses with lysosomes and its content is being degraded (Figure 3).

One of the most described pathways of mitophagy is the PINK1/Parkin-mediated autophagy [195]; notably, mutations in the Parkin and PINK1 genes are the most common causes of recessive forms of Parkinson's Disease characterized by early onset [196, 197]. Specifically, the PINK1 gene encodes a serine/threonine kinase, which localizes in the outer membrane of depolarized mitochondria. Other forms of PINK1 that are processed by the rhomboid protease PARL can be found in the inner mitochondrial membrane or in the cytosol [198, 199]. Following PARL cleavage, PINK1 is degraded by mitochondrial proteases, and thus in most cells the levels of PINK1 that associate with mitochondria are undetectable or very low [198]. Parkin encodes a cytosolic E3 ubiquitin ligase that mediates polyubiquitination of its substrates (e.g., Mfn1 and Mfn2) on the outer mitochondrial membrane and initiates the mitophagic process [200, 201]. The ubiquitinated mitochondrial proteins can be degraded by either the autophagy machinery or the ubiquitin-proteasome system [202, 203].* Drosophila* studies have shown that PINK1 and Parkin act in the same pathway since expression of Parkin in a background of mutated PINK1 in flies partially rescued the phenotype [204–206].

Mitochondrial depolarization stabilizes PINK1 on the outer mitochondrial membrane; this event directly phosphorylates Parkin and induces its recruitment in the mitochondria. Parkin then ubiquitinates the fusion proteins Mfn1 and Mfn2 and the proteins involved in mitochondrial trafficking, Miro1 and Miro2 [200, 203]. Moreover, the increased levels of Parkin induce ubiquitination of other outer mitochondrial membrane proteins, such as the voltage-dependent anion channel (VDAC) and the components of the TOM mitochondrial translocase complex [200, 203, 206–208]. Interestingly, Mfn1, Mfn2, and VDAC knockout mice still undergo mitophagy suggesting that the role of these proteins in mitophagy induction needs to be further investigated [209, 210]. After Parkin-mediated ubiquitination of the outer mitochondrial membrane proteins, the selective autophagy adapter protein p62/SQSTM1 (Sequestosome 1) is recruited to mitochondria where it is thought to promote autophagy due to its capacity to directly interact with the LC3 receptor (Figure 3) [132, 211, 212]. Mitochondrial depolarization with carbonyl cyanide m-chlorophenylhydrazone (CCCP) treatment induces the accumulation of histone deacetylase 6 (HDAC6) in the mitochondrial outer membrane. p62/SQSTM1 and HDAC6 interact with Ambra1 and Beclin1, prompting the accumulation of the autophagosome to mitochondria [213, 214]; interestingly, studies in p62 knockout mice showed that p62 also mediates mitochondrial perinuclear clustering [212]. Recently, optineurin was found to be recruited to mitochondria in order to induce autophagosome formation around the damaged mitochondria via LC3 receptor [215].

Several studies link the PINK1/Parkin pathway to mitochondrial dynamics, namely, fission/fusion and motility. Specifically, PINK1 phosphorylates the fusion protein Mfn2 and this event, likely, induces recruitment of Parkin to mitochondria [216]. Mitofusins not only are substrates for PINK1 and Parkin but also can regulate their proteasomal turnovers through ubiquitination [217]. Furthermore, studies in mammalian cells have shown that overexpression of PINK1 induced mitochondrial elongation, while its knockdown promoted fragmentation [218, 219]. Increasing fusion events prevent the degradation of mitochondria by starvation-induced autophagy [220]. Recently, several evidences link mitochondrial fission events and mitophagy. The yeast homolog of Drp1, Dnm1, is required in certain mitophagy types. Thus mitochondria fragmentation induced by fission probably facilitates autophagosome engulfment [221].

Like mitofusins, Miro is also phosphorylated by PINK1 and ubiquitinated by Parkin. Parkin-dependent ubiquitination of Miro leads to proteasomal degradation and arrest of mitochondrial motility [222]. Moreover, it was shown that Mfn2 interacts with Miro in the mitochondria axonal transport [223]; indeed, PINK1 and Parkin can affect Miro directly or indirectly by targeting Mfn2 to degradation. Also, genome screening studies have identified additional PINK1/Parkin regulators like SMURF1 (SMAD specific E3 ubiquitin protein ligase 1), ATPIF1/IF1 (ATPase inhibitory factor 1), and TOMM7 which, likely, promote autophagy [224–226].

Additional mechanisms that affect the PINK1/Parkin-mediated mitophagy include the activity of PI3K/AKT pathway in starvation conditions; this event attenuates mitophagy. On the other hand, mitophagy is enhanced by accumulation of unfolded proteins in the mitochondrial matrix or downregulation of the LONP1 peptidase (Human LON protease homolog) [227, 228]. Interestingly, lack of the PINK1 and Parkin yeast homologs does not seem to affect the removal of damaged mitochondria by autophagy. In another stressful condition, namely, nitrogen starvation, the Atg32/Atg11 complex recruits the fission machinery to interact with the Dnm1 protein and to induce mitochondria degradation by autophagy [221].

Despite the growing knowledge about the PINK1/Parkin pathway involvement in mitophagy, most of the studies are performed in models with altered expression of Parkin. The majority of the cell systems are treated with CCCP, which totally depolarize the mitochondrial membrane resulting in Parkin overexpression [229]; it is therefore still unclear to what extent endogenous Parkin mediates autophagy [230]. In fact, Parkin knockout mice presented failure of heart functionality and mitochondria aggregation, while no recruitment of Parkin on mitochondria was observed when it was overexpressed [231].

Although in the most studies mitophagy was induced artificially, in a recent work it was shown that constitutive mitophagy, which requires PINK1 and Parkin, occurs in mouse primary hippocampal neurons without mitochondrial membrane depolarization or drug treatment [232].

In addition, loss of Drp1 leads to mitochondria ubiquitination, accumulation of damaged mitochondria, and p62 mitochondrial targeting, independently from Parkin [233, 234]. Indeed, it seems that there must be additional proteins that regulate mitophagy in a Parkin-independent way. In line with this assumption, studies in* Drosophila* showed that the mitochondrial ubiquitin ligase 1 (MUL1) totally rescued the phenotype of PINK1/Parkin loss of function [235]. Other autophagy receptor proteins which have been shown to induce mitophagy in a Parkin-independent pathway include BNIP3 (BCL-2/Adenovirus E1B 19 kDa Interacting Protein 3) and NIX (also called BNIP3L) that interact with the LC3 receptor and induce mitophagy in hypoxic conditions. Deletion of either BINP3 or NIX alone does not affect mitophagy, suggesting that both proteins are needed to promote mitophagy [236]. NIX null mice showed retention of mitochondria in erythrocytes and, likely, NIX is not required for mitophagy induction but rather acts as a receptor for targeting autophagosomes to mitochondria (e.g., like the Atg32 in yeast) [237]. Another protein that can induce Parkin-independent mitophagy is Cardiolipin, a phospholipid dimer of the mitochondrial inner membrane. Induced mitochondrial damage leads to translocation of Cardiolipin in the outer membrane followed by increased LC3 colocalization with damaged mitochondria [238].

Recently, two new Parkin-independent pathways have been described. Targeted overexpression of AMBRA1 at the mitochondrial outer membrane induces autophagy in both Parkin-dependent and Parkin-independent ways [239]. Similarly, a new Parkin-independent role of PINK1 in mitophagy was proposed [240]. Specifically, it was shown that PINK1 phosphorylation of ubiquitin molecules on mitochondrial membrane acts as an autophagic signal. PINK1, in the absence of Parkin, recruits NDP52 (also known as CALCOCO2, Calcium binding and Coil-Coil domain protein 2) and optineurin, but not p62, to mitochondria to activate (Parkin-independent) mitophagy. According to this new model phosphorylation of ubiquitins by PINK1 is needed to recruit Parkin and autophagy receptors to mitochondria. In the absence of Parkin, PINK1 induces blind levels of mitophagy using the relatively low basal ubiquitin levels on mitochondria. In the presence of Parkin the signal is amplified, since Parkin generates more ubiquitin chains on mitochondria which are subsequently phosphorylated by PINK1 enhancing the rate and levels of clearance [240] (Figure 3).

An additional mechanism for the removal of damaged mitochondria is the formation of mitochondria-derived vesicles (MDV) [241]. MDV are cargo-selective vesicles released from mitochondria which fuse with lysosomes and undergo hydrolytic degradation. The MDV formation is induced by increased ROS species and does not require mitochondrial depolarization [241]. Although MDV-mediated degradation is independent of the canonical autophagic proteins LC3 and ATG5, it still requires a PINK/Parkin functional pathway [241].

Overall, mitophagy is an important mitochondrial quality control mechanism that effectively removes damaged mitochondria in order to prevent oxidative stress and cellular death. Considering the growing number of proteins involved in this process, the variation in mitophagic events, and its functional implication in ageing and age-related diseases, further detailed studies are needed to clarify and better understand this highly dynamic process.

## 6. Mitochondria and Ageing

Ageing is a physiological process that occurs despite the presence of complex pathways of maintenance, defense, and repair, and it has been correlated with a number of diseases including cancer, neurodegenerative diseases, diabetes, and heart failure; notably, there are no evolutionary selected “gerontogenes” which function to cause ageing, while (among others) ageing correlates with increased proteome instability which leads to irreversible cellular damage and dysfunction [4, 5, 242–246].

In relation to mitochondria, generation of a transgenic mouse model with mutated mtDNA provided the first genetic evidence that mutated mtDNA leads to premature ageing [247]. Moreover, mitochondria are the primary source of ROS which seem to accumulate during ageing [248, 249], due to an (among others) age-related increase of mtDNA mutations which then increase ROS levels by affecting the respiratory chain [250–252]. Ageing decline of mitochondrial functionality is also associated with mitochondrial morphological alterations and decrease of mitochondria numbers [253, 254], as well as with a decrease of autophagic activity and reduced mitochondrial biogenesis [255, 256]; therefore, mitochondria dynamics seems to have an important role in the progression of ageing. Reduced expression of Mfn2 and Drp1 genes in the skeletal muscle of aged individuals suggested an impairment of fusion/fission event in skeletal muscle fibers; this could lead to loss of muscle strength and mass with age [257]. In support, reduced fission in mouse model is associated with muscle atrophy [258].

Several studies have shown that caloric or dietary reduction increases lifespan [259–261]. Insulin/IGF-1 signaling (IIS) and target of rapamycin (TOR) signaling pathways are the two main nutrient-sensing pathways that have been linked to lifespan regulation [262–264]. Studies in mice have shown that caloric restriction increases mitochondrial respiration and mitochondria biogenesis through sirtuin 1 activation [13, 265, 266]; in support, a diet that is rich in compounds that are known to impair mitochondrial functionality and accumulate during ageing, namely, advanced glycation end products (AGEs) or lipofuscin [267–269], reduced lifespan and affected proteasome activities in* Drosophila* [270]. Thus, endogenous or exogenous factors which affect the mitochondria bioenergetics and/or biogenesis have a direct impact on ageing and, likely, on age-related diseases (see below).

## 7. Mitochondria Quality Control and Cancer

The “Warburg Effect” was proposed by Warburg and suggested that cancer cells have a metabolic shift toward aerobic glycolysis (rather than oxidative phosphorylation), reduced mitochondrial respiration, and functionally altered mitochondria in order to provide sufficient energy for their growth [16]; nevertheless, in many types of cancer, tumor cells still depend on energy production by mitochondria and thus do not suppress mitochondrial bioenergetics.

Accumulation of mtDNA mutations along with increased levels of ROS (that enhance mutation on the mitochondrial genome) have been described as promoting factors of tumorigenesis [271–274]; in addition, many mtDNA mutations that associate with tumorigenesis were shown to inhibit OXPHOS [275, 276]. In support, exchange of mtDNA with pathogenic or normal mtDNA in cancer cells resulted in alterations of cancer cell phenotypes [277, 278], further underlying the important role of mtDNA in tumorigenesis. In addition, mutation of the mitochondrial transcription factor A (TFAM) in some colorectal cancers was associated with mtDNA depletion, while its overexpression promoted cell proliferation [279, 280].

Increased ROS levels, which largely originate from dysfunctional mitochondria, promote the activation of a number of transcription factors, including nuclear respiratory factor 2 (NRF2), the nuclear factor-kappa beta (NF-*κ*B), and the Hypoxia Inducible Factor 1*α* (HIF1*α*). The transcription factors NRF1 and NRF2 prompt the expression of the nuclear genes encoding subunits of the mitochondrial respiratory chain complexes and NRF2 activation increases synthesis of anabolic enzymes, NADPH production, and purine biosynthesis which all correlate with increased tumor growth [281]. Moreover, according to recent findings, NRF1 and NRF2 seem to be important in mitochondrial biogenesis and respiratory chain reactions [282, 283]; likewise, the role of NF-*κ*B in tumorigenesis and mitochondria functionality has been adequately demonstrated in several studies [284]. NRF2 has been implicated in promotion of tumorigenesis by suppressing ROS levels and NRF2 knockout mice showed high levels of ROS and decreased tumorigenesis [285, 286]. In addition, Nrf2 was recently identified as a candidate transcriptional regulator of proteasome genes. Proteasome dysfunction in* Drosophila* induces high levels of reactive oxygen species that originated from malfunctioning mitochondria, triggering an Nrf2-dependent upregulation of the proteasome subunits [287].

The high proliferative rate of tumor cells leads (among others) to insufficient blood supply with nutrient and oxygen. Therefore hypoxic conditions are a feature of tumor cells* in vivo*. Hypoxia increases ROS levels that further stabilize HIF*α* transcription factor subunits so the cell can adapt to reduced oxygen levels [288, 289]. HIF1*α* binds to genomic hypoxia-responsive elements promoting the expression of a large number of genes including glycolytic enzymes and pyruvate dehydrogenase kinase-1 (PDK1 inhibits conversion of pyruvate to acetyl CoA) and it also inhibits LON protease that (among others) degrades COX4-1 subunit [62, 290, 291]. In addition, LON is thought to play an important role in metabolic reprogramming and cellular senescence and it also increases the oncogenic potential of tumor cells [67, 292, 293].

Since increased ROS levels are a common feature of cancer cells therapeutic approaches that aim to decrease intracellular ROS levels have been considered as a possible method to inhibit cancer growth [294–296]. However, these treatments can also affect normal cells where ROS play an import functional role (e.g., macrophages) [249]. Another reason why the use of these approaches has not been so successful is the fact that mitochondrial ROS are important signaling molecules and potent mitogens. Moreover, recently, it was shown that increased oxidative stress suppressed metastasis on melanoma cells [297], suggesting that increased levels of ROS may have an antioncogenic role; in line with this notion, antioxidants are frequently upregulated in cancer cells in order to suppress oxidative stress-mediated apoptotic effects and reduced proliferation [298].

Another factor being activated during tumorigenesis is peroxisome proliferator-activated receptor gamma coactivator 1-alpha (PGC-1*α*; a member of the PGC-1 family of coactivators) which is considered a key regulator of mitochondrial biogenesis and respiration. The PGC-1 family members potentiate the activity of other transcription factors and PGC-1*α* interacts with NRF1 and PPAR*α* [299, 300]. PGC-1*α* can also reduce the generation of mitochondrial ROS and it also regulates the mitochondrial fusion machinery by activating Mfn2 [301]. High expression levels of PGC-1*α* were found to be induced by the melanocyte-specific transcription factor (MITF) in melanoma cells, while growth and progression of these melanoma cells were strongly dependent on PGC-1*α* expression levels [302]. Moreover, it was recently reported that Parkin regulates the expression of PGC-1*α*. Activation of Parkin promotes degradation of PARIS (a KRAB and zinc finger protein) which normally inhibits expression of PGC-1*α* by binding to insulin response sequences in the PGC-1*α* promoter [303].

Mitochondrial biogenesis is also controlled by the c-Myc protooncogene. c-Myc induces the activation of the PGC-1*β* factor; on the other hand, mitochondrial biogenesis is inhibited when HIF1 factors promote degradation of c-Myc [304].

Several tumor types have altered levels of mitophagy-related proteins. Parkin levels are downregulated in a number of different tumors, including ovarian, lung, and breast cancer, sporadic colorectal cancer, hepatocellular carcinoma, and pancreatic tumors, while PINK1 is overexpressed in adrenocortical (ACT) tumors [305–308]. Reportedly, the BNIP3 and NIX mitophagy genes are upregulated in different premalignant stages of some tumor types, while their expression is suppressed in invasive and malignant cancers [309–311]. Loss of BNIP3 probably leads to genome instability in pancreatic cancer, likely, due to increased ROS levels [312].

Finally, alterations of the mitochondrial fusion/fission rate and machinery have been also observed in tumors. More specifically, several reports indicate that fission (linked to upregulation of Drp1 or downregulation of Mfn2) is increased in a variety of tumors, including lung cancer and invasive breast carcinoma [313, 314]. Also, hypoxic conditions enhance the rate of fission events by modulating Drp1 activity, while enhancement of fission in U251 human glioblastoma cells promoted tumor cell migration [315, 316].

## 8. Mitochondria Quality Control and Neurodegeneration

Neuronal cells function and survival strongly depend on proper mitochondria functionality and activity, since axonal transport, neurotransmitter releasing, and ionic gradient can be severely impaired by dysfunctional mitochondria [317, 318]. In line with these facts a number of neurological disorders, including Alzheimer's Disease (AD), Parkinson's Disease (PD), and Huntington's Disease (HD), as well as amyotrophic lateral sclerosis (ALS), have been associated with the quality control of this organelle and the proteins involved. In support, mutations at PINK1 and Parkin genes are the most prevalent in patients with autosomal recessive PD early onset [319].* Drosophila* PINK1 or Parkin loss of function exhibits muscle and neuron degenerations which are highly reminiscent of Parkinson's Disease [204]. Moreover, the MitoPark mouse model (an animal model of Parkinson's Disease) is characterized by mitochondria fragmentation and respiratory deficiency in dopaminergic neurons [231]. Nevertheless, and despite the plethora of information which is available about these proteins, it still remains relatively unclear how PINK1/Parkin mitochondrial dysfunction leads to neurodegeneration. That is, likely, due to the fact that a great number of the studies about PINK1/Parkin are performed in cellular systems after artificially induced mitochondrial damage and depolarization leading probably to mitochondria and cell conditions which are significantly different, or at least with reduced similarity, with those found in neurological diseases. A main feature of PD is the Lewy body formation. The nonmitochondrial protein of alpha-synuclein is the major component of Lewy bodies [319]. Alpha-synuclein is degraded by proteasome and alpha-synuclein aggregates impaired normal proteasomal function [320, 321]; moreover, patients with sporadic or familial forms of PD display altered proteasome function [321].

AD is characterized by the formation of characteristic amyloid-*β* (A*β*) plaques and neurofibrillary tangles (as result of the association of mainly fibrillar forms of A*β* and tau protein with microtubules), impaired mitochondrial trafficking, and increased ROS levels [322]. Amyloid-*β*-peptide can accumulate at mitochondria and probably interacts with Drp1, while AD cellular models present decreased levels of Drp1 protein and increased expression of the Fis1 counterpart [323, 324]. UPS dysfunction seems to be also involved in AD disease, since the amyloid-*β* plaques formation impairs normal proteasomal function; this effect further fuels the formation of neurofibrillary tangles [325, 326].

Several other neurodegeneration diseases are associated with mitochondrial proteins dysfunction. Impaired fusion of the inner membrane due to Opal mutations leads to dominant optic atrophy, whether mutation of the outer membrane fusion protein Mfn2 is linked to peripheral neuropathy 30 Charcot-Marie-Tooth type 2A [176, 327]. Furthermore, mouse knockouts of Mfn1/2 and Opa1 genes result in embryonic lethality [170, 328], while mutations of the m-AAA subunit paraplegin lead to an autosomal recessive form of hereditary spastic paraplegia [79–81]. Mutations of the m-AAA subunit AFGL32 are linked to spinocerebellar ataxia [329], while mutations of Hsp60 in humans have been implicated in the pathogenesis of hereditary spastic paraplegia [330]. Finally, a mouse model lacking HtrA2 displayed neurodegeneration and PD-like phenotypes and missense mutations of HtrA2 have been reported in sporadic cases of PD [94, 331, 332].

The association of mitochondria function and dynamics with these neurological disorders highlights the central role of this organelle in proper functionality of neuronal cells. Besides mitochondria studies, even more data describe the UPS dysfunction in neurodegeneration disorders [333–335]. Nevertheless, more detailed research is required in relation to the functional involvement of UPS in neuronal cells function and how this system interacts with mitochondria in neuronal tissue.

## 9. Concluding Remarks

The vital role of mitochondria in cellular homeodynamics is clearly reflected in the severe effects of mitochondrial dysfunction on cellular functionality and human health, ageing, and age-related diseases (e.g., cancer or neurodegeneration).

Despite the growing knowledge about the molecular mechanisms that impose on mitochondrial function and structural preservation several controversial questions remain to be answered. For example, although mitochondria dysfunction (or altered function) seems to be a common feature in both neurodegeneration and cancer, the disease-specific alterations that determine the fate of the disorder need further detailed investigation efforts. In this line of research, the identification of the mitochondrial maintenance and/or signaling pathways that are specifically implicated in malignancy or neurodegeneration will, likely, reveal new disease-specific therapeutic approaches; similar efforts should aim at identifying how loss of mitostasis impacts on the progression of ageing.

An additional topic of exciting future research should of course relate to the identification of the molecular pathways that regulate the intense cross talk between the proteostatic and mitostatic modules in the young and aged somatic and reproductive tissues and how deterioration of one pathway affects the functionality of the other; these efforts will be particularly relevant given the UPS involvement in mitochondrial quality control and functionality and* vice versa*.

Moreover, the triggering event(s) that modulate the activation of the UPS systemic responses or mitophagy following mitochondrial damage clearly need further investigations. Most likely, the disrupted balance of ATP production (that initiates significant metabolic alterations) along with membrane depolarization and ROS accumulation influences the equilibrium between the selective removal of mitochondria by mitophagy or UPS-mediated degradation of damaged mitochondrial proteins.

Finally, another aspect of significant importance relates to the question whether UPS is also involved in the degradation of proteins of the internal mitochondrial compartments.

A better understanding of the mechanisms that regulate mitochondria quality control and their interconnection with the proteostasis modules (e.g., UPS) is relevant for human health since, besides the basic knowledge, mitochondria and proteasomes apart from impacting organismal healthspan are, likely, key therapeutic targets in main age-related diseases including cancer and neurodegeneration.

## Abbreviations

ALS: Autophagy Lysosome System
OXPHOS: Oxidative phosphorylation
PDR: Proteome Damage Responses
PN: Proteostasis network
ROS: Reactive oxygen species
UPS: Ubiquitin-proteasome-system
UPR^mt^: Mitochondrial Unfolded Protein Response
ULK1: Unc-51-Like Kinase 1 protein
RB1CC1: RB1-inducibile Coiled-Coil 1
Keap1: Kelch-like ECH-associated protein 1
Nrf2: NF-E2-related factor 2
Drp1: Dynamin-related protein 1
OPA1: Optic Atrophy 1
PARL: Presenilin-Associated Rhomboid-Like
Miro: Mitochondrial Rho GTPase
AMBRA1: Activating autophagy/beclin-1 regulator 1
p62/SQSTM1: Sequestosome 1
AD: Alzheimer's Disease
PD: Parkinson's Disease.
